# Diagnostic Potential of Apparent Diffusion Coefficient-Based Lymph Node Classification in Breast Cancer Patients Undergoing [^18^F]FDG-PET/MRI

**DOI:** 10.3390/diagnostics16111712

**Published:** 2026-06-02

**Authors:** Helena A. Peters, Marie Scheuer, Daniel Weiss, Matthias Boschheidgen, Vivien Lorena Ivan, Frederic Dietzel, Svjetlana Mohrmann, Eugen Ruckhäberle, Ken Herrmann, Harald H. Quick, Aleksandar Milosevic, Peter Minko, Julian Kirchner, Lale Umutlu, Gerald Antoch, Kai Jannusch

**Affiliations:** 1 Department of Diagnostic and Interventional Radiology, Medical Faculty, University Düsseldorf, 40225 Düsseldorf, Germany; ingemarie.scheuer@med.uni-duesseldorf.de (M.S.); danielarvid.weiss@med.uni-duesseldorf.de (D.W.); matthias.boschheidgen@med.uni-duesseldorf.de (M.B.); vivienlorena.ivan@med.uni-duesseldorf.de (V.L.I.); frederic.dietzel@med.uni-duesseldorf.de (F.D.); peter.minko@med.uni-duesseldorf.de (P.M.); julian.kirchner@med.uni-duesseldorf.de (J.K.); antoch@med.uni-duesseldorf.de (G.A.); kai.jannusch@med.uni-duesseldorf.de (K.J.); 2 Department of Gynecology, Medical Faculty, University Düsseldorf, 40225 Düsseldorf, Germany; mohrmann@med.uni-duesseldorf.de (S.M.); eugen.ruckhaeberle@med.uni-duesseldorf.de (E.R.); 3 Department of Nuclear Medicine, University Hospital Essen, University of Duisburg-Essen and German Cancer Consortium (DKTK), 45147 Essen, Germany; ken.herrmann@uk-essen.de; 4 High-Field and Hybrid MR Imaging, University Hospital Essen, 45147 Essen, Germany; harald.quick@uk-essen.de; 5 Erwin L. Hahn Institute for MR Imaging, University of Duisburg-Essen, 45141 Essen, Germany; 6 Department of Diagnostic and Interventional Radiology and Neuroradiology, University Hospital Essen, University of Duisburg-Essen, 45147 Essen, Germany; aleksandar.milosevic@uk-essen.de (A.M.); lale.umutlu@uk-essen.de (L.U.); 7 Center for Integrated Oncology Aachen Bonn Cologne Düsseldorf (CIO ABCD), 40225 Düsseldorf, Germany

**Keywords:** breast cancer, PET/MRI, apparent diffusion coefficient, lymph node metastases

## Abstract

**Background/Objectives**: To evaluate the diagnostic potential of apparent diffusion coefficient (ADC) values for classifying lymph nodes as benign or malignant in breast cancer patients undergoing [^18^F]FDG-PET/MRI staging. **Methods**: Mean ADC values and short-axis diameters (±standard deviation) of 199 thoracic lymph nodes in 113 newly diagnosed breast cancer patients were retrospectively analyzed. All patients underwent [^18^F]FDG-PET/MRI staging, between July 2017 and June 2021. A node-by-node comparison was performed with respect to pathological node status. Nodal FDG uptake in whole-body [^18^F]FDG-PET/MRI served as reference standard for nodal malignancy. Group comparison using Mann–Whitney U test, receiver operating characteristic curve (ROC) analysis and diagnostic performance were calculated. *p* values below 0.05 were defined as statistically significant. Confidence intervals (CI; 95%) were calculated. **Results**: Ninety-three lymph nodes were FDG-negative while 106 lymph nodes were FDG-positive. FDG-negative lymph nodes had significantly lower short-axis diameters ((5.1 ± 1.5 mm versus 12.3 ± 5.3 mm); *p* < 0.01; U: 405.50; Z: −11.24). ADC values were significantly lower in FDG-positive lymph nodes (0.72 ± 0.14 × 10^−3^ mm^2^/s) than in FDG-negative lymph nodes ((1.18 ± 0.18 × 10^−3^ mm^2^/s); *p* < 0.01; U: 173.00; Z: −11.80). ROC analysis and Youden’s index revealed an ADC cut-off of 0.95 × 10^−3^ mm^2^/s (AUC: 0.98; *p* < 0.01; 95% CI: 0.96–1.01). According to the calculated cut-off, sensitivity, specificity, and accuracy of ADC values for differentiating FDG-negative from FDG-positive lymph nodes were 98%, 97% and 97%, respectively. **Conclusions**: ADC values derived from MRI were significantly associated with FDG uptake in this retrospective cohort and may serve as a complementary imaging biomarker for lymph node characterization.

## 1. Introduction

Breast cancer (BC) is the most common malignancy among women worldwide, with nearly 2.3 million new cases diagnosed in 2022 and ranks fourth in global cancer-related mortality [1,2]. Apart from tumor biology and distant metastases, appearance of lymph node metastases is one of the key predictors of prognosis in breast cancer patients and significantly influences therapeutic decision-making, including the extent of axillary surgery or the need for regional radiotherapy [3,4]. The 5-year survival rate for patients without lymph node metastases is 96%, consistently declining with increased number of metastatic lymph nodes [5,6]. Therefore, precise pre-therapeutic assessment of locoregional lymph node status becomes even more important in terms of treatment planning.

Accurate axillary staging is a critical component in the management of breast cancer, as it guides therapeutic decision-making and prognosis [7]. Current guidelines recommend ultrasound as the primary imaging modality for axillary evaluation, followed by sentinel lymph node biopsy (SLNB) as the gold standard in patients with clinically node-negative disease and negative axillary ultrasound. Axillary lymph node dissection (ALND) is now reserved for patients with confirmed macrometastases in the sentinel node or with clinically or radiologically suspicious lymph nodes [8,9,10,11]. Despite these advances, both SLNB and ALND remain invasive procedures associated with short- and long-term complications, underscoring the need for reliable non-invasive alternatives [10,12].

For systemic staging, computed tomography (CT) of the chest and abdomen in combination with bone scintigraphy has traditionally been recommended to detect distant metastases [13]. However, CT lacks sensitivity for axillary lymph node characterization [14,15]. In contrast, [^18^F]fluorodeoxyglucose positron emission tomography/computed tomography ([^18^F]FDG-PET/CT) has emerged as a valuable tool for detecting both nodal and distant metastases and recent evidence suggests a higher accuracy compared with conventional staging [16]. Whole-body magnetic resonance imaging (MRI), although rarely used in clinical routine, can be combined with dedicated breast MRI and provides well-recognized advantages in assessing parenchymal organs [17]. More recently, [^18^F]FDG-PET/MRI has demonstrated superior diagnostic performance compared with conventional methods, highlighting their potential role in improving preoperative axillary staging [18,19]. Within this evolving landscape, functional imaging biomarkers derived from diffusion-weighted MRI are of growing interest.

Diffusion-weighted imaging (DWI) is an MRI technique that assesses tissue microstructure by evaluating the diffusion of water molecules. In areas of high cellular density, such as malignant tumors, water movement is significantly restricted, whereas diffusion is facilitated in tissues with lower cellularity or structural damage. These diffusion properties can be quantitatively measured using the apparent diffusion coefficient (ADC) providing an objective parameter for tissue characterization. The ADC is a quantitative parameter that reflects the magnitude of water molecule diffusion within tissue. It is expressed in units of mm^2^/s and calculated from the signal attenuation at different b-values using a monoexponential model [20,21]. DWI offers valuable, non-invasive insights into tissue properties without requiring the administration of contrast agents, making it particularly advantageous for patients with contraindications to contrast media, such as during pregnancy or in cases of impaired renal function [22,23]. In recent years, DWI has been increasingly incorporated into breast MRI protocols to improve diagnostic capabilities [24,25].

Thus, this retrospective study aimed to evaluate the ability of MRI-derived ADC values to differentiate between malignant and benign lymph nodes in breast cancer patients, using [^18^F]FDG-PET as the reference standard for nodal malignancy. By focusing on the diagnostic performance of ADC, our findings provide insight into the potential of diffusion-weighted MRI as a non-invasive approach for lymph node assessment, supporting clinical decision-making and might reduce the need for invasive procedures.

## 2. Materials and Methods

### 2.1. Patients

This retrospective study was approved by the institutional review boards of the University of Duisburg-Essen (approval number: 17-7396-B0) and the University of Düsseldorf (approval number: 6040R). All procedures were carried out in accordance with the Declaration of Helsinki (1964 and later amendments). Written informed consent was obtained from all participants prior to study inclusion.

Between July 2017 and June 2021, a retrospective cohort of patients with newly diagnosed, treatment-naïve breast cancer was assembled at the University Hospital Düsseldorf and the University Hospital Essen, Germany. Only patients considered to be at increased risk for distant metastatic disease were eligible for inclusion, according to predefined criteria.

For staging purposes, all patients underwent combined [^18^F]FDG-PET/MRI. Inclusion criteria were defined as follows: (i) primary breast cancer staged T2 or higher without prior therapy; (ii) triple-negative breast cancer of any tumor size at initial diagnosis; or (iii) biologically high-risk tumors characterized by Ki-67 > 14%, grade 3 histology, or HER2 overexpression. Patients were excluded if they were pregnant or lactating, had a history of malignancy within the previous five years, or presented with contraindications to MRI or gadolinium-based contrast agents.

### 2.2. [^18^F]FDG-PET/MRI

All imaging examinations were performed using an integrated 3.0 Tesla PET/MRI system (Biograph mMR; Siemens Healthineers AG, Forchheim, Germany). Patients were examined in the supine position, and imaging started at a mean interval of 108 ± 20 min after intravenous injection of [^18^F]FDG (weight-adapted dose of 4 MBq/kg; mean administered activity 216 ± 50 MBq). Patients were required to fast for at least 6 h before tracer administration, and blood glucose levels were confirmed to be below 150 mg/dL prior to injection.

The field of view (FOV) included the body volume from head to the mid-thigh using a dedicated 16-channel head-and-neck radiofrequency (RF) coil, a 24-channel spine-array RF coil and up to five 6-channel flex body coils. The examination was performed in supine position, head-first with arms next to the body.

PET data were acquired simultaneously with MRI in 3 min bed positions across four to five bed positions (axial field of view: 25.8 cm; matrix: 344 × 344). Image reconstruction was performed using an ordered subset expectation maximization (OSEM) algorithm (three iterations, 21 subsets), followed by Gaussian post-filtering with 4 mm full width at half maximum and reconstruction into a 344 × 344 matrix. For MR-based attenuation correction of the patient tissues, a two-point (fat, water) coronal 3D-Dixon-VIBE sequence was acquired to generate a four-compartment model (background air, lungs, fat, muscle) [26,27].

The whole-body MRI protocol consisted of the following sequences:

1. Axial T2-weighted HASTE sequence acquired in breath-hold technique (slice thickness 7 mm; TE 97 ms; TR 1500 ms; turbo factor 194; FOV 400 mm; phase FOV 75%; matrix 320 × 240; in-plane resolution 1.3 × 1.3 mm; acquisition time 0:47 min per bed position).
2. Axial diffusion-weighted imaging (EPI) performed under free breathing (slice thickness 5 mm; TR 7400 ms; TE 72 ms; b-values 0, 500, 1000 s/mm^2^; matrix 160 × 90; FOV 400 × 315 mm; GRAPPA factor 2; in-plane resolution 2.6 × 2.6 mm; acquisition time 2:06 min per bed position). ADC maps were automatically generated using a dedicated workstation.
3. Fat-suppressed contrast-enhanced 3D T1-weighted VIBE sequence acquired during breath-hold (slice thickness 3 mm; TE 1.53 ms; TR 3.64 ms; flip angle 9°; FOV 400 × 280 mm; matrix 512 × 384; in-plane resolution 0.7 × 0.7 mm; acquisition time 0:19 min per bed position).

### 2.3. Image Analysis

All images were reviewed independently and in random order by two radiologists experienced in hybrid imaging and one nuclear medicine specialist, using an OsiriX workstation (Pixmeo SARL, Bernex, Switzerland). A four-week wash-out period separated the two reading sessions to minimize recall bias. Discrepant findings were adjudicated in a joint consensus meeting. Formal interobserver agreement statistics were not performed.

For each patient, thoracic lymph node stations including axillary, mediastinal, internal mammary, supraclavicular/cervical, and pectoral nodes were assessed. Lymph nodes with a short-axis diameter ≥ 3 mm and sufficient image quality for reliable ADC assessment were included, irrespective of FDG uptake status. In patients with multiple lymph nodes, more than one assessable node could be included in the analysis. Morphological parameters recorded included maximal short-axis diameter (in mm), effacement of fatty hilus (yes or no) and nodal shape (rounded, oval, or irregular).

**Figure 1:**
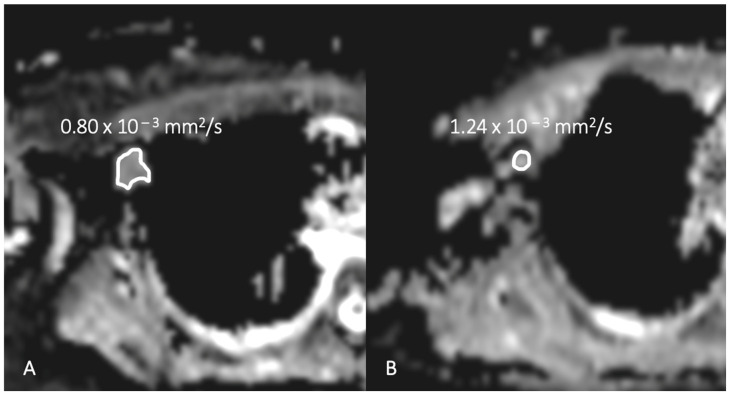
Representative examples of ADC value assessment. (**A**,**B**) Manual regions of interest (ROIs) were drawn on the gray-scale ADC maps along the inner margin of the lymph node on the central slice with the greatest diameter.

For the assessment of ADC values, a manually drawn region of interest (ROI) was placed on the gray-scale ADC map on the central slice of each lymph node with the greatest diameter, covering nearly the entire inner margin of the lymph node. ADC measurements were performed on a single slice and not as a volumetric assessment. ROI placement was performed using the combined evaluation of DWI, fused [^18^F]FDG-PET/MRI images, and corresponding contrast-enhanced T1-weighted MRI sequences. The ROI was carefully positioned to avoid inclusion of adjacent fatty tissue and to exclude areas suggestive of hemorrhage or necrosis based on their morphological appearance and signal characteristics, thereby minimizing partial volume effects. Qualitative assessment of signal intensity on gray-scale DWI images was not included in this study. ADC values were reported in units of ×10^−3^ mm^2^/s throughout the manuscript. For comparison with studies using ×10^−6^ mm^2^/s, a value of 0.72 × 10^−3^ mm^2^/s corresponds to 720 × 10^−6^ mm^2^/s. A representative example of ADC measurement and ROI placement is shown in Figure 1.

**Figure 2:**
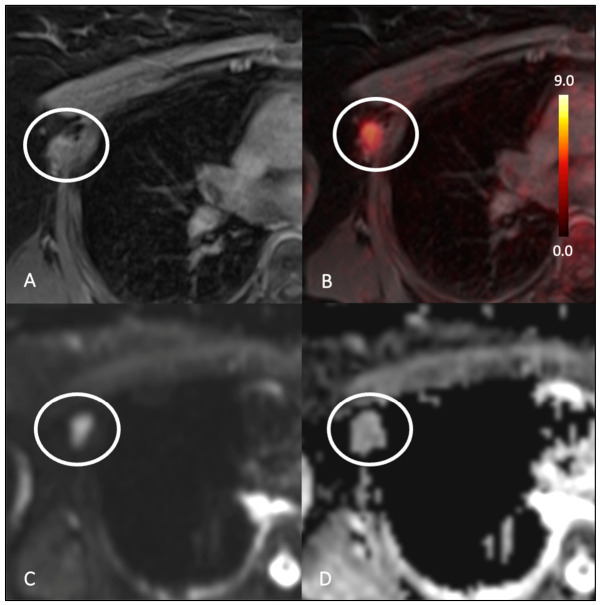
Right axillary lymph node metastasis in a breast cancer patient (tumor stage according to TNM classification: cT2 N1 M0). Gadolinium-enhanced T1 image (**A**) and fusion image of [^18^F]FDG-PET/MRI (**B**) showing strong, focal tracer signal (FDG+). Focal area of high signal intensity on b1000 DWI image (**C**) and with low ADC value (0.80 × 10^−3^ mm^2^/s) on the ADC map (**D**).

**Figure 3:**
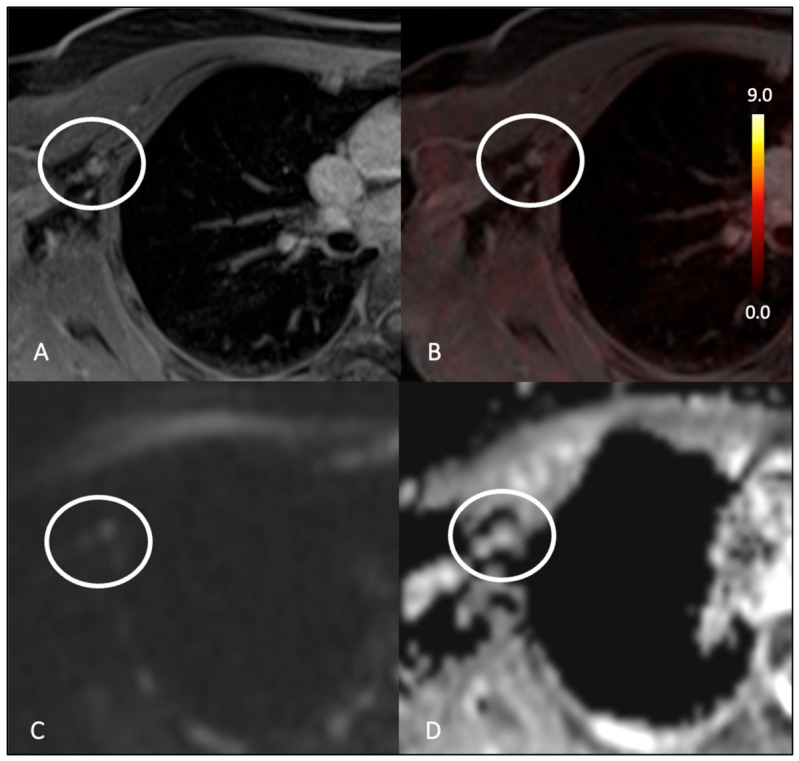
Right axillary benign lymph node in a breast cancer patient (tumor stage according to TNM classification: cT2 N0 M0). Gadolinium-enhanced T1 image (**A**) and fusion image of [^18^F]FDG-PET/MRI (**B**) showing no focal tracer signal (FDG−). Focal area of moderate signal intensity on b1000 DWI image (**C**) and with moderate ADC value (1.24 × 10^−3^ mm^2^/s) on the ADC map (**D**).

On [^18^F]FDG-PET/MRI, lymph nodes were visually classified as FDG-positive or FDG-negative based on focal [^18^F]FDG uptake relative to the surrounding background activity and adjacent lymph nodes. Visual assessment was performed within the integrated hybrid [^18^F]FDG-PET/MRI examination and served as the imaging-based reference standard for nodal malignancy assessment. Example images of a lymph node metastasis and a benign lymph node are presented in Figure 2 and Figure 3.

### 2.4. Statistics

Statistical analyses were conducted with SPSS Statistics, version 31.0 (IBM Corp., Armonk, New York, USA). Continuous variables were reported as mean ± standard deviation (SD), while non-normally distributed data were documented using median and interquartile range (IQR, 25th–75th percentile). Lymph node characterization based on the average short-axis diameter and mean ADC values, assessed independently by two blinded readers, was used to differentiate between FDG-avid and non-FDG-avid nodes. Group differences were assessed with the Mann–Whitney U test. An optimal ADC cut-off was determined from receiver operating characteristic (ROC) curves by maximizing Youden’s index. Diagnostic performance was expressed as the area under the ROC curve (AUC). In addition, sensitivity, specificity, and overall diagnostic accuracy were calculated. A multivariate logistic regression analysis (odds ratio) was performed to assess the risk of FDG positivity based on lymph node morphology (presence of a fatty hilum and nodal shape) in combination with an ADC value. A two-sided *p* < 0.05 was considered statistically significant and confidence intervals were examined (CI; 95%).

## 3. Results

### 3.1. Patient Characteristics

A total of 113 women with newly diagnosed breast cancer were retrospectively analyzed. The mean age was 50 ± 12 years (Table 1). Patient demographics and lymph node characteristics and localization are presented in Table 1. All patients underwent [^18^F]FDG-PET/MRI.

### 3.2. Thoracic Lymph Nodes

**Figure 4:**
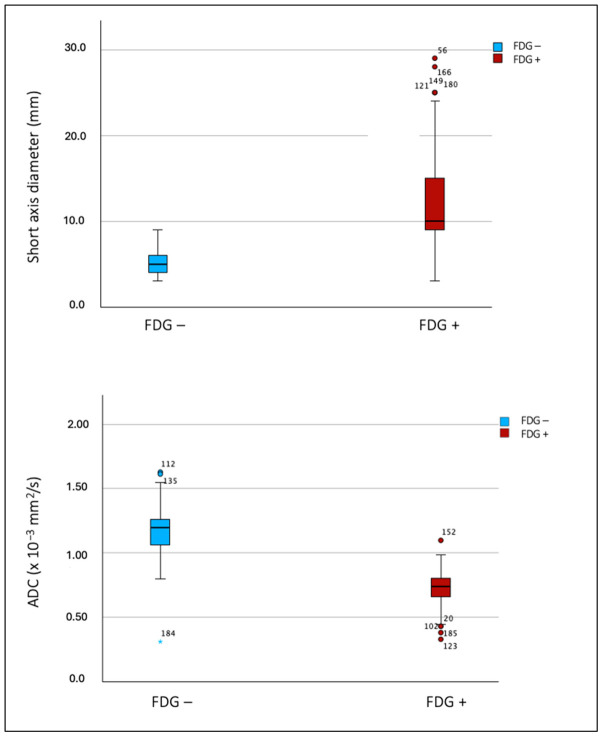
Boxplot showing the distribution of short-axis diameter and ADC for lymph nodes with (FDG+) and without tracer accumulation (FDG−) in [^18^F]FDG-PET/MRI. The asterisk (*) indicates an extreme outlier, defined as a value located more than three interquartile ranges (IQRs) from the edge of the box. Circles indicate outliers located between 1.5 and 3 IQRs from the box.

**Figure 5:**
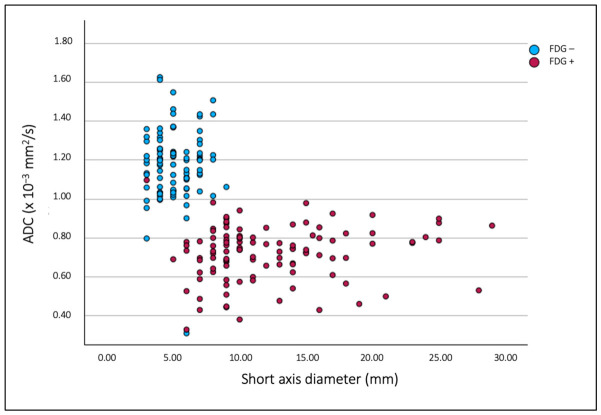
Scatterplot illustrating the differentiation of FDG-positive (FDG+) and FDG-negative (FDG−) lymph nodes by ADC values and short-axis diameter. Unlike the short-axis diameter, the ADC enables reliable discrimination between FDG-positive and FDG-negative lymph nodes.

Based on the reference standard, 93/199 lymph nodes were classified as FDG-negative and 106/199 as FDG-positive. The mean short-axis diameter and mean ADC measurements of the two blinded raters were used to compare FDG-positive and FDG-negative nodes. The mean short-axis diameter was 12.3 ± 5.3 mm for FDG-positive and 5.1 ± 1.5 mm for FDG-negative lymph nodes, as shown in Table 1 and in Figure 4. A significant difference in short-axis diameter was observed between FDG-positive and FDG-negative lymph nodes (*p* < 0.01; U: 405.50; Z: −11.24). FDG-positive lymph nodes showed a significantly lower ADC value (0.72 ± 0.14 × 10^−3^ mm^2^/s) compared to FDG-negative lymph nodes (1.18 ± 0.18 × 10^−3^ mm^2^/s; *p* < 0.01; U: 173.00; Z: −11.80), as shown in Table 2 and in Figure 4. ADC values appeared to be a more robust parameter than the short-axis diameter for distinguishing FDG-positive from FDG-negative lymph nodes, as FDG-positive nodes exhibited consistently lower ADCs with minimal overlap between groups (Figure 5).

**Figure 6:**
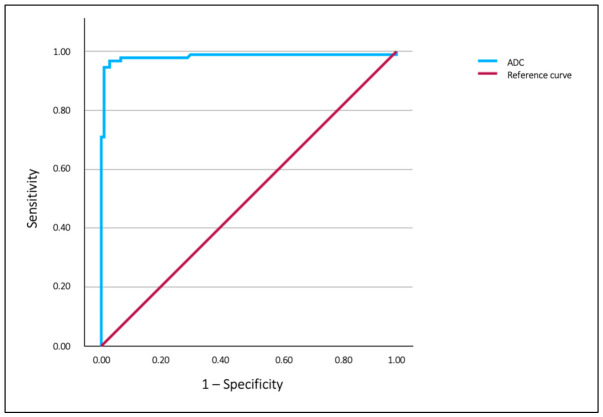
Receiver operating characteristic (ROC) curve analysis of the apparent diffusion coefficient (ADC) for differentiating FDG-positive from FDG-negative lymph nodes. The ADC-based model demonstrated high diagnostic accuracy, with an area under the curve (AUC: 0.98; *p* < 0.01; 95% CI: 0.96–1.00).

Using ROC analysis and maximizing Youden’s index, a cut-off value was determined to differentiate between FDG-positive and FDG-negative nodes (Figure 6). The optimal cut-off value was of 0.95 × 10^−3^ mm^2^/s with a large AUC of 0.98 (*p* < 0.01; 95% CI: 0.96–1.00).

### 3.3. Morphology of Thoracic Lymph Nodes

The morphology of thoracic lymph nodes was analyzed, focusing on the presence of a fatty hilum (yes/no) and nodal configuration (round, oval, or irregular). Among FDG-positive lymph nodes, the majority exhibited an oval shape (52/106, 49%), 41/106 (39%) were round, and 13/106 (12%) were irregular. Most FDG-positive lymph nodes (101/106, 95%) did not show a morphologically visible fatty hilum, whereas 5/106 (5%) displayed a fatty hilum despite FDG positivity.

**Figure 7:**
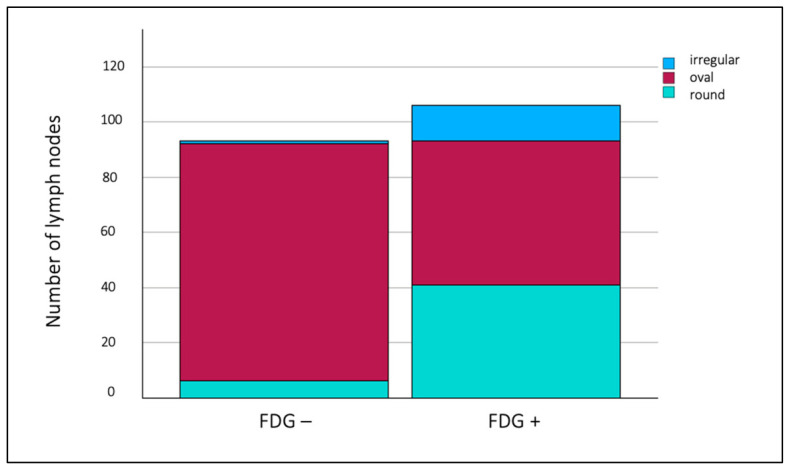
Bar chart illustrating the distribution of nodal shapes (oval, round, irregular) in FDG-positive (FDG+) and FDG-negative (FDG−) lymph nodes. FDG-positive lymph nodes showed a higher proportion of round configurations compared to FDG-negative lymph nodes.

In FDG-negative lymph nodes, most were oval in shape (87/93, 94%), 5/93 (5.4%) were round, and 1/93 (1%) was irregular (Figure 7). Regarding the fatty hilum, 86/93 (93%) had a visible fatty hilum, while in 7/93 (8%) it was not clearly delineated.

Multivariate regression analysis revealed that the absence of a fatty hilum was strongly associated with FDG positivity (OR = 86.18; 95% CI: 30.10–246.74; *p* < 0.01). In addition, a round nodal shape (OR = 4.40; 95% CI: 1.16–16.64; *p* < 0.05) and an ADC value lower than 0.95 × 10^−3^ mm^2^/s (OR = 3.10; 95% CI: 1.11–8.71; *p* < 0.05) were independently associated with an increased likelihood of FDG positivity.

## 4. Discussion

This study demonstrates that the use of ADC values in [^18^F]FDG-PET/MRI may help differentiate benign from malignant thoracic lymph nodes in breast cancer patients, with FDG uptake serving as the internal reference standard for nodal malignancy. Assessment of ADC values may support more accurate N-staging on MRI in breast cancer patients.

We observed a strong correlation between ADC values and FDG uptake, with FDG-avid lymph node metastases showing significantly lower ADC values than FDG-negative nodes. These findings highlight the potential of ADC as a promising non-invasive imaging biomarker, and the identification of an effective ADC cut-off value further supports its possible clinical applicability for preoperative lymph node assessment. Nevertheless, FDG uptake may also occur in inflammatory or reactive lymph nodes and should therefore be interpreted cautiously in the absence of histopathological confirmation [28,29,30].

Metastatic lymph nodes are characterized by increased cellularity and higher metabolic activity, resulting in restricted diffusion on DWI and consequently lower ADC values, while simultaneously showing elevated FDG uptake. In contrast, benign lymph nodes generally exhibit higher ADC values and lower metabolic activity [31]. This physiological relationship between tissue composition, diffusion restriction, and glucose metabolism provides the biological rationale for the diagnostic potential of ADC values in lymph node assessment [32,33,34]. Our findings are consistent with this biological rationale, demonstrating significantly higher ADC values in FDG-negative lymph nodes compared with FDG-positive lymph nodes in breast cancer patients. While previous studies predominantly relied on histopathological confirmation for lymph node classification [5,35,36,37], our study used FDG uptake derived from hybrid [^18^F]FDG-PET/MRI as an imaging-based reference standard. This approach enabled combined anatomical and metabolic characterization of thoracic lymph nodes in a non-invasive setting, although it does not replace histopathological validation. The use of FDG uptake as an imaging-based reference standard was chosen because precise one-to-one correlation between imaging findings and histopathology is often technically challenging in clinical routine, particularly for numerous small thoracic lymph nodes.

Despite these methodological differences, previous studies, such as those by Guvenc et al. and Razek et al., reported findings comparable to ours [37,38]. In their study, the ADC value of histopathologically confirmed malignant lymph nodes was significantly lower (0.89 × 10^−3^ mm^2^/s and 1.08 × 10^−3^ mm^2^/s) compared to benign lymph nodes (1.41 × 10^−3^ mm^2^/s and 1.15 × 10^−3^ mm^2^/s). The systemic review by Azizzadeh et al. also demonstrated a significant difference in ADC values between benign and malignant lymph nodes. The pooled ADC value for benign lymph nodes was 1.272 × 10^−3^ mm^2^/s, whereas malignant lymph nodes showed a lower mean ADC value of 0.874 × 10^−3^ mm^2^/s [36]. Similar to these studies and review using histopathologically confirmed lymph nodes, we also identified a significant ADC cut-off value. However, in our study, this threshold was derived from the differentiation between FDG-negative and FDG-positive lymph nodes based on [^18^F]FDG-PET/MRI findings. In our cohort, the optimal ADC cut-off value was 0.95 × 10^−3^ mm^2^/s. According to this threshold, the sensitivity, specificity, and accuracy of ADC values for differentiating benign from malignant lymph nodes were 98%, 97%, and 97%, respectively, within the context of our imaging-based reference standard. The cut-off values reported in the literature vary across a range around 1.00 × 10^−3^ mm^2^/s. For example, Guvenc et al. reported a cut-off of 0.985 × 10^−3^ mm^2^/s, with a sensitivity of 83% and specificity of 98% [37], while Fardanesh et al. reported a cut-off of 1.004 × 10^−3^ mm^2^/s, yielding an accuracy of 75%, sensitivity of 71%, specificity of 79%, positive predictive value of 77%, and negative predictive value of 74% [39]. In the study by Luo et al., a cut-off value was determined using the ratio between the ADC of the lymph node and the ADC of the breast lesion [40]. However, this approach may not always be clinically applicable, particularly in patients who have already undergone surgery. Nonetheless, it can be suitable for pre-therapeutic imaging. Differences in reported ADC thresholds between studies may be related to variations in MRI field strength, applied b-values, ROI placement techniques, patient populations, and the respective reference standards used for lymph node classification [41,42]. These methodological differences highlight the need for greater standardization of ADC acquisition and analysis protocols to improve comparability across studies and to support future clinical implementation.

Overall, it is important to note that our study did not limit the analysis to axillary lymph nodes but included all thoracic lymph nodes. However, it should be acknowledged that the majority of evaluated lymph nodes were located in the axillary region, whereas the numbers of other thoracic lymph node stations were comparatively small. Therefore, although the inclusion of all thoracic lymph node regions may broaden the scope of the analysis, the present findings predominantly reflect axillary lymph node assessment and should be interpreted cautiously regarding generalization to other thoracic nodal stations.

Given the limitations of MRI alone in reliably differentiating between benign and malignant lymph nodes, particularly in borderline or indeterminate cases, hybrid imaging techniques such as [^18^F]FDG-PET/MRI may offer enhanced diagnostic accuracy. [^18^F]FDG-PET/MRI combines the functional information of metabolic activity from PET with the high soft tissue contrast of MRI [17,18,19]. Thus, potentially serving as a more robust and non-invasive tool for lymph node classification. Nevertheless, histopathological confirmation remains the diagnostic gold standard for nodal malignancy assessment [43]. However, biopsy procedures are also subject to sampling errors and reported false-negative rates ranging from 5% to 24% [44,45].

Overall, as demonstrated in our study and supported by previous research [36,37,38], DWI-derived ADC values may allow reliable characterization of thoracic and axillary lymph nodes without the need for radiation exposure or contrast media. This makes it an attractive tool for clinical application. Its value is particularly evident in patient populations where contrast administration is contraindicated or should be minimized, such as those with impaired renal function, known allergies to gadolinium-based agents, or during pregnancy. Importantly, the status of axillary lymph nodes remains one of the most critical prognostic factors in breast cancer, as it directly determines staging, treatment strategies, and long-term outcomes [46]. Therefore, reliable and non-invasive methods to detect lymph node metastases are highly desirable. ADC-based lymph node assessment may represent a promising adjunctive imaging approach with the potential to support preoperative nodal evaluation. Future prospective studies with histopathological correlation are warranted to further investigate whether ADC-based assessment could contribute to reducing the need for invasive procedures such as SLNB or ALND in selected patients. Since SLNB is currently the gold standard for clinically node-negative patients with negative axillary ultrasound, implementing non-invasive imaging approaches preoperatively may provide significant advantages, including reduced costs and shorter operation times.

It should also be noted that, in addition to ADC values, nodal shape and the absence of a fatty hilum significantly influenced the results in our study, with lymph nodes lacking a fatty hilum and exhibiting a round shape showing a higher likelihood of FDG positivity. This finding is consistent with previous studies reporting that the absence of a fatty hilum and a round configuration are highly specific features of malignant lymph nodes [28,47]. Although ADC demonstrated excellent overall discriminative performance in ROC analysis, ADC values remained independently associated with FDG positivity even after adjustment for established morphologic features. The corresponding odds ratio in the multivariate regression model was only moderate, likely because it was derived from a dichotomized ADC threshold after adjustment for additional morphologic covariates. Therefore, the moderate odds ratio does not contradict the high AUC observed for ADC values. In this context, structured reporting systems such as the Node-RADS (Node Reporting and Data System) classification are already established and have demonstrated promising high diagnostic performance across various cancer types by integrating morphological criteria, including nodal shape and hilum characteristics, into a standardized risk stratification framework [48]. It would therefore be very interesting to consider incorporating ADC values as an additional functional parameter within this existing system. Doing so could further refine MRI-based lymph node assessment, complement the established Node-RADS criteria, and enhance quantitative evaluation, potentially improving diagnostic confidence and preoperative risk stratification in diverse oncologic settings.

This study has several limitations. Most importantly, FDG uptake of the lymph nodes was used as the reference standard instead of surgical findings or histopathology. Although [^18^F]FDG-PET/MRI is a valuable and widely used imaging modality in oncologic staging, increased FDG uptake may also occur in inflammatory or reactive lymph nodes, potentially reducing specificity [28,29,49]. Consequently, the reported diagnostic performance metrics, including sensitivity and specificity, may have been overestimated due to the imperfect imaging-based reference standard. Therefore, the present findings should be interpreted cautiously and require further validation in prospective studies with histopathological correlation. Nevertheless, image interpretation was performed in a specialized oncologic imaging setting with substantial clinical expertise, which may have reduced the likelihood of misclassification. In addition, thoracic lymph nodes are often small and numerous, making precise one-to-one correlation between imaging and histopathological findings technically challenging in clinical routine.

Furthermore, partial volume effects and MRI-related artifacts may have influenced ADC measurements. In addition, the spatial resolution of the diffusion-weighted imaging protocol and the use of manually drawn single-slice ROIs rather than volumetric assessment may have introduced measurement variability, particularly in small lymph nodes. These effects may have been especially relevant in lymph nodes close to the spatial resolution limits of PET imaging, while differences in magnetic field strength and b-values may limit comparability across datasets. Future studies may benefit from advanced imaging techniques aimed at reducing image distortion and improving signal-to-noise ratio. The study population mainly consisted of patients with higher-risk breast cancer characteristics, including larger tumor burden and biologically aggressive tumor subtypes, which may limit generalizability to lower-risk populations. Moreover, the very high diagnostic performance observed in this retrospective single-center cohort may partly reflect optimistic estimation of diagnostic accuracy, particularly in the absence of external validation. The optimal ADC cut-off value was derived and evaluated within the same retrospective cohort without internal resampling or cross-validation, which may have further contributed to overestimation of diagnostic performance. Formal interobserver agreement statistics were not performed, as image evaluation was based on consensus assessment within the exploratory study design. Therefore, reproducibility of ADC measurements and morphologic assessment could not be formally quantified. Finally, multiple lymph nodes from individual patients were included in the analysis, which may have introduced intra-patient clustering effects and potentially led to overestimation of statistical significance. Future studies should incorporate statistical approaches accounting for non-independent observations, such as mixed effects models or generalized estimating equations.

## 5. Conclusions

Our findings highlight the potential of MRI-derived ADC measurements for the non-invasive characterization of lymph nodes in newly diagnosed breast cancer patients. Using FDG uptake as an imaging-based reference standard, ADC values accurately differentiated benign from malignant lymph nodes. ADC-based assessment may thus support preoperative nodal evaluation, though prospective histopathological validation is needed to confirm the clinical applicability of these findings.

## Figures and Tables

**Table 1:** Patient demographics and lymph node characteristics and localization.

Patients	Data
Total patients	113
Sex (female)	113
Mean age ± SD (years)	50 ± 12
Mean number of lymph nodes examined per patient	1.8 ± 0.8
**Lymph Nodes**	**Data**
Total lymph nodes	199
FDG+	106
FDG−	93
**Mean Short-Axis Diameter ± SD (mm)**	
All lymph nodes	9.0 ± 5.4 mm
FDG+	12.3 ± 5.3 mm
FDG−	5.1 ± 1.5 mm
Short-axis diameter ≤ 10 mm	147
Short-axis diameter > 10 mm	52
**Localization**	
Axillary	180
Pectoral	10
Supraclavicular/cervical	7
Mammaria interna	2

**Notes:** Data are *n*, except for age (years), mean number of lymph nodes examined per patient, and short-axis diameters (mm), which are given as mean ± standard deviation. FDG: [^18^F]Fluorodeoxyglucose.

**Table 2:** ADC values of FDG-positive (FDG+) and FDG-negative (FDG−) lymph nodes assessed using the Mann–Whitney U test and optimal ADC cut-off for differentiation.

FDG Uptake	
FDG+ (mean ADC ± SD)	0.72 ± 0.14 × 10^−3^ mm^2^/s
FDG− (mean ADC ± SD)	1.18 ± 0.18 × 10^−3^ mm^2^/s
*p*	<0.01
U	173.00
Z	−11.80
**ROC Analysis**	
ADC cut-off	0.95 × 10^−3^ mm^2^/s
AUC	0.98, *p* < 0.01; 95% CI: 0.96–1.00
Sensitivity	98%
Specificity	97%
Accuracy	97%

**Notes:** FDG: [^18^F]fluorodeoxyglucose.; ADC: apparent diffusion coefficient; CI: confidence interval.

## Data Availability

The datasets generated during and/or analyzed during the current study are available from the corresponding author on reasonable request.

## Abbreviations

The following abbreviations are used in this manuscript:

ADC	apparent diffusion coefficient
ALND	axillary lymph node dissection
BC	breast cancer
CT	computer tomography
CI	confidence interval
DWI	diffusion-weighted imaging
EPI	echo-planar imaging
[^18^F]FDG-PET/CT	[^18^F]fluorodeoxyglucose positron emission tomography/computed tomography
[^18^F]FDG-PET/MRI	[^18^F]fluorodeoxyglucose positron emission tomography/magnetic resonance imaging
FWHM	full width at half maximum
FOV	field of view
HASTE	half-Fourier acquisition single-shot turbo spin echo
IQR	interquartile range
MRI	magnetic resonance imaging
Node-RADS	Node-RADS (Node Reporting and Data System)
OSEM	ordered subset expectation maximization
RF	radiofrequency
ROC	receiver operating characteristic
ROI	region of interest
SD	standard deviation
SLNB	sentinel lymph node biopsy
VIBE	volumetric interpolated breath-hold examination
